# Prevalence and correlates of HIV infection among people who use drugs in Cambodia: a cross-sectional survey using respondent driven sampling method

**DOI:** 10.1186/s12879-019-4154-5

**Published:** 2019-06-11

**Authors:** Sovannary Tuot, Gitau Mburu, Phalkun Mun, Pheak Chhoun, Navy Chann, Kiesha Prem, Siyan Yi

**Affiliations:** 1 KHANA Center for Population Health Research, Phnom Penh, Cambodia; 2 Division of Health Research, Lancaster University, Lancaster, UK; 3 National Center for HIV/AIDS, Dermatology and STD, Phnom Penh, Cambodia; 4 Center for Global Health Research, Touro University California, Vallejo, USA; 5 Saw Swee Hock School of Public Health, National University of Singapore and National University Health System, Tahir Foundation Building, 12 Science Drive 2, #10-01, Singapore, 117549 Singapore

**Keywords:** Drug abuse, HIV risk, Harm reduction, Key population, National survey, Cambodia

## Abstract

**Background:**

Most of studies on the relationship between drug use and HIV have focused largely on people who inject drugs. Non-injecting drug use is much more common than injecting drug use, and although it can also predispose people to HIV infection, it is not widely explored. We therefore conducted this study to explore the prevalence of HIV and identify risk factors for HIV infection among people who use non-injecting drugs (PWUD) in Cambodia.

**Methods:**

This cross-sectional study was conducted in 2017. The Respondent Driven Sampling method was used to recruit the study participants who were interviewed face-to-face using a structured questionnaire. Blood samples were collected for HIV and syphilis testing. A multivariable logistic regression analysis was conducted to identify risk factors associated with HIV infection.

**Results:**

In total, 1367 PWUD were included in this study, whose mean age was 28.0 (SD = 7.7) years. The majority (95.1%) of the participants used methamphetamine. The prevalence of HIV was 5.7, and 35.2% of the identified HIV-positive PWUD were not aware of their status prior to the survey. After adjustment for other covariates, HIV infection remained significantly associated with being in the age group of ≥35 (AOR = 2.34, 95% CI = 1.04–6.11), having lower level of formal education of ≤ 6 years (AOR = 2.26, 95% CI = 1.04–5.15), living on the streets (AOR = 2.82, 95% CI = 1.10–7.23), perception that their HIV risk was higher as compared to that of the general population (AOR = 3.18, 95% CI = 1.27–8.62), having used injecting drugs in lifetime (AOR = 3.8, 95% CI = 1.36–4.56), and having cuts or sores around the genital area in the past 12 months (AOR = 3.42, 95% CI = 1.09–6.33).

**Conclusions:**

The prevalence of HIV among PWUD in this study was more than 10 times higher than the prevalence in the general adult population. The findings reveal a higher vulnerability to HIV infection among specific sub-populations of PWUD, such as those who are homeless, who may benefit from tailored interventions that respond to their specific needs. To enhance HIV case finding, stratification of PWUD to facilitate HIV risk profiling based on socio-economic profiles and drug injection history is recommended.

## Background

Illicit drug use is a significant global health problem. Annually, a quarter of a billion people use at least one illicit drug, with cannabis, amphetamines, cocaine, and heroin being the most common [1]. Evidence from a recent systematic review suggested that illicit drug dependence directly accounted for 20 million disability-adjusted life years (DALYs) in 2010, representing 0.8% of global all-cause DALYs [2]. This burden of disease is exacerbated by the intersection between the use of illicit drugs, poor mental health, and suicides [2, 3].

In Cambodia, people who use drugs (PWUD), particularly people who inject drugs (PWID), are considered an important group for HIV prevention and harm reduction programs. The current Cambodia 3.0 framework aims to eliminate new HIV infections in the country by 2020, by accelerating prevention among all key populations including PWUD and PWID, female entertainment workers, men who have sex with men, and transgender women [4]. To achieve this goal, a key emphasis in the national response is an aspiration to strengthen the availability of data and strategic information related to the HIV epidemic among the key populations [5].

In relation to illicit drug use, however, greater emphasis in Cambodia has been placed on PWID with limited attention to people who use drugs via non-injecting modes [5, 6]. While injection of illicit drugs clearly increases the risk of HIV and viral hepatitis infections [7, 8], ignoring non-injecting consumption of illicit drugs will prevent the achievement of the Cambodian national goal of eliminating HIV infection. We argue that focusing on wider substance abuse is important for two reasons. First, the use of illicit drugs, particularly stimulants, has been shown to increase risk of HIV, regardless of injection [7]. Secondly, the use of illicit drugs such as cannabis, amphetamines, cocaine, or heroin frequently occurs alongside the consumption of other substances such as alcohol and tobacco [9, 10], which have their own impacts on health and health behaviors. For instance, the concomitant use of alcohol alongside the illicit drugs has been shown to further compromise HIV prevention by reducing condom use [11].

In the Cambodian context, PWUD are specifically defined as people who have used any types of illicit drugs, as specified in the Cambodian Law on Control of Drugs, via any route of administration other than injection in the past 12 months [12]. In 2012, it was estimated that there were 13,000 PWUD and 1300 PWID nationally [4]. A more recent size estimation exercise conducted alongside this national survey concluded that there were 22,374 PWUD and 4136 PWID in the country in 2017 [13]. In both of these estimates, PWID were not subsumed under PWUD. Despite the large numbers of PWUD, studies focusing on this population are limited. Rare studies have shown that PWUD in Cambodia have an increased risk of mental health problems [14]. However, further data related to this population are needed to inform HIV prevention and other public health interventions. Accordingly, characterization of the extent and risk factors of non-injecting drug use was purposely emphasized in this Integrated Biological and Behavioral Survey (IBBS 2017). In this paper, we reported the prevalence of HIV and factors associated with HIV infection among PWUD in Cambodia. We also discussed the potential implications of these findings on HIV prevention policies and programs.

## Methods

### Study design, sites, and participants

This cross-sectional IBBS 2017 among PWUD and PWID in Cambodia was conducted from June to December 2017 in the capital city of Phnom Penh and 11 other major provinces purposively selected by the research team and approved by the National Center for HIV/AIDS, Dermatology and STD (NCHADS) and National Authority for Combating Drugs (NACD). These study sites were selected based on results from a feasibility assessment conducted as part of the study designing and protocol development. About 70% of the Cambodian population resides in the 12 provinces out of 25 provinces in Cambodia. The 12 provinces included: Banteay Meanchey, Battambang, Kampong Cham, Kampong Chhnang, Kampong Speu, Kandal, Phnom Penh, Preah Sihanouk, Prey Veng, Siem Reap, Svay Rieng, and Tbong Khmum. According to the national epidemiological data from NCHADS and size estimation studies, the selected sites consisted of 21 operational districts with a high burden of HIV and drug use, i.e. more than 80% of estimated drug use in Cambodia. To be included in the study, an individual must: (1) be at least 18 years old; (2) have a valid study coupon; (3) have not participated in this survey before; (4) meet the criteria defining PWUD; and (5) be able and willing to provide a written informed consent.

### Sample size and sampling procedures

The estimated PWUD population size of 13,000 [15] and the assumption that the prevalence of HIV would be lower compared to the prevalence in the national survey in 2012, justified on the basis of the scaled up provision of community-based harm reduction services in recent years [4, 6], informed the sample size calculation for this survey. Using an estimated HIV prevalence of 3.5%, a margin error of 1.5%, a confidence interval of 95%, a response rate of 90%, and a design effect of 1.5, we derived a minimum sample size of 1390 for this study. The calculated sample size was further stratified by study sites, and roughly 15% of the estimated PWUD in each site were recruited. Data collection was conducted in 21 locations: six locations in Phnom Penh and 15 locations in the remaining 11 provinces. The precise number of the selected locations was determined based on the required sample size in each study site. The Respondent Driven Sampling (RDS) method was used to recruit study participants.

The RDS was implemented in five stages. First, eligible four seeds who were well connected to other PWUD in each location were invited to participate in the survey with support from a non-governmental organization (NGO) working in the area. A written informed consent was obtained from them. Second, each seed was given a Personal Identification Number (PIN) and enrolled as a participant. Third, each seed received three coupons and were asked to refer three additional PWUD. They would receive US$2 for a successful referral. Each seed was expected to extend up to three to six “recruitment waves” at each site. If the initial seeds did not recruit participants or if the enrollment has been halted because all recruitment chains have “dried up” (i.e. stopped recruiting), additional seeds would be selected based on the inclusion criteria. Finally, recruited individuals were provided the same opportunity as seeds to recruit other PWUD. In total, 84 seeds were selected for the entire survey.

### Data collection training

All data collection team members participated in a three-day training workshop, which covered a review of the study protocol, data collection methods, tool pretesting, and reflection. The training also included interview techniques, confidentiality, and privacy and provided the study teams with an opportunity to practice questionnaire administration. The training facilitators explained the study protocol and tools in details during the sessions to ensure that all team members were thoroughly familiar with it. A regular daily review session with data collection teams were conducted during the survey period to review progress and communicate any issues encountered during the data collection.

### Data collection procedures

#### HIV and syphilis test

HIV and syphilis screening were performed by a laboratory technician using SD Bioline HIV/Syphilis Duo test. A non-HIV reactive result established that an individual was not HIV-infected. In the case of an HIV reactive result from the SD Bioline HIV/Syphilis Duo test, a confirmatory test was conducted on site using HIV 1/2 STAT-PAK® Assay. The participants were given an option to receive their screening results verbally in a post-test counseling session after the questionnaire interview. A follow-up on newly detected HIV-infected individuals was pursued closely by a local NGO working in the area. Participants received a token gift, which costed approximately US$5, to compensate them for their time and transport expenses.

#### Questionnaire development and measures

A structured questionnaire was developed using standardized tools adapted from previous studies among HIV key populations in Cambodia and the most recent Cambodia Demographic and Health Survey [14–18]. The questionnaire was initially designed in English and then translated into Khmer, the national language of Cambodia. Another translator then back-translated it into English to ensure that the “content and spirit” of every original item were maintained. A consultative meeting was held with representatives of PWUD and key stakeholders working on HIV and harm reduction in Cambodia. A pilot study was conducted with 20 PWUD in Phnom Penh, who were later excluded from the main study.

Socio-demographic characteristics included geographical location of the study site (urban or rural), age (continuous), gender, level of education attained (continuous), average monthly income in the past 6 months (continuous), living situations, and employment status. Regarding drug use, we collected information on types of illicit drugs most commonly used and frequency of use in the past 3 months. We also collected information regarding exposure to community-based HIV, harm reduction, and other related services in the past 6 months. To measure HIV risks, participants were asked about their sexual behaviors in the past 3 months including number of partners and condom use with commercial (defined as a partner with whom the participant had sex in exchange for money or goods) and non-commercial partners in the past 3 months. Other collected information included HIV testing history, STI symptoms, and care seeking behaviors as well as the use of other substances (tobacco and alcohol) in the past 3 months.

### Data analyses

The prevalence of HIV was calculated by dividing the total number of participants tested positive for HIV with the total number of participants tested. In bivariate analyses, we compared characteristics and behavioral variables of participants who were HIV positive to those of participants who were HIV negative. Chi-square test (or Fisher’s exact test for an expected cell value of ≤5) was used for categorical variables, and Student’s *t*-test or Mann-Whitney U test for continuous variables. To facilitate the analyses, age, level of education, and income were transformed into categorical variables. A multivariable logistic regression model was constructed to identify independent factors associated with HIV infection. Variables with a significance level of *p* < 0.05 in the bivariate analyses were simultaneously included in the model. Age, gender, level of education, and income were included in the model regardless of the significance level in bivariate analyses because they have been found associated with HIV infection in previous studies.

### Ethical considerations

This study was approved by the National Ethics Committee for Health Research (NECHR) of the Ministry of Health, Cambodia (No. 193 NECHR). Privacy and confidentiality of the participants were protected by conducting the data collection in a private room and removing all personal identifiers from the study documents. Participants were fully informed about the voluntary nature of the study as well as risks and benefits they may expect from their participation in the study. A written informed consent was obtained from each participant.

## Results

### Socio-demographic characteristics

This study included 1367 PWUD with a mean age of 28.0 years (SD = 7.7). As shown in Tables 1, 87.9% of the participants were residing in urban communities, and 39.6% were female. Almost half (47.8%) had never been married, and 52.9% had attained only primary education. While 48.2% reported living with their parents or relatives, 16.8% were living on the streets. The most common jobs included a laborer or farmer (38.6%) and an entertainment worker (16.6%), while 11.6% were unemployed. The majority of the participants (77.0%) reported an average monthly income in the past 6 months of <US$200. A significantly higher proportion of HIV-positive participants were female (59.0% vs. 38.4%), were in the age group of ≥35 (35.9% vs. 18.3%), had completed only primary education (74.4% vs. 51.6%), lived on the streets (28.2% vs. 16.1%), and had the main job as a sex worker (14.1% vs. 3.3%) compared to HIV-negative group.

**Table 1:** Socio-demographic characteristics of HIV-positive and HIV-negative PWUD

Socio-demographic characteristics	Total (*n* = 1367)	HIV test result		
		Positive (*n* = 78)	Negative (*n* = 1289)	
	*n* (%)	*n* (%)	*n* (%)	*P*-value^*^
Living in an urban community	1201 (87.9)	73 (93.6)	1128 (87.5)	0.11
Female gender	541 (39.6)	46 (59.0)	495 (38.4)	< 0.001
Age group				< 0.001
18–24	519 (38.0)	18 (23.1)	501 (38.9)	
25–34	583 (42.7)	32 (41.0)	551 (40.4)	
≥ 35	263 (19.3)	28 (35.9)	235 (18.3)	
Current marital status				0.14
Never married	651 (47.8)	29 (37.2)	622 (48.5)	
Married	498 (36.6)	33 (42.3)	465 (36.2)	
Widowed/divorced/separated	212 (15.6)	16 (20.5)	196 (15.3)	
Level of formal education attained				< 0.001
Primary (0–6 years)	722 (52.9)	58 (74.4)	664 (51.6)	
Secondary school (7–9 years)	287 (28.4)	13 (16.7)	374 (29.1)	
High school or higher (≥10 years)	213 (15.6)	7 (9.0)	248 (19.3)	
Living situation				< 0.001
In the street (homeless)	229 (16.8)	22 (28.2)	207 (16.1)	
With parents or relatives	659 (48.2)	20 (25.6)	639 (49.6)	
In own dwelling	322 (23.6)	27 (34.6)	295 (22.9)	
With friends	157 (11.5)	9 (11.5)	148 (11.5)	
Main occupation				0.004
Unemployed	158 (11.6)	9 (11.5)	149 (11.6)	
Hair dresser/beautician	76 (5.6)	4 (5.1)	72 (5.6)	
Office worker	63 (4.6)	4 (5.1)	59 (4.6)	
Laborer/farmer	528 (38.6)	22 (28.2)	506 (39.3)	
Self-employed	152 (11.1)	7 (9.0)	145 (11.2)	
Entertainment worker^†^	227 (16.6)	15 (19.2)	212 (16.4)	
Sex worker	53 (3.9)	11 (14.1)	42 (3.3)	
Student	38 (2.8)	0 (0.0)	38 (2.9)	
Other	72 (5.3)	6 (7.7)	66 (5.1)	
Average monthly income in the past 6 months (US$)				0.57
< 100	493 (36.2)	31 (39.7)	462 (36.0)	
100–199	556 (40.8)	27 (34.6)	529 (41.2)	
200–299	199 (14.6)	11 (14.1)	188 (14.6)	
≥ 300	115 (8.4)	9 (11.5)	106 (8.2)	

HIV, human immunodeficiency virus; PWUD, people who use drugs; US$, United States dollar

^*^Chi-square (or Fisher’s exact test when a cell count was smaller than 5) was used

^†^Entertainment workers, often women, work in entertainment establishments such as karaoke bars, beer gardens, and massage parlors

### Prevalence of HIV

The prevalence of HIV was 5.7% (95% CI = 4.6–7.1%). Provinces in the top-five highest prevalence of HIV were Preah Sihanouk (13.6%), Battambang (9.3%), Banteay Meanchey (8.6%), Siem Reap (6.0%), and Kampong Chhnang (4.6%). No newly-identified HIV-positive participants were found in Kampong Cham and Prey Veng. More than one-third (35.2%) of the HIV-positive cases were not aware of their status prior to the study. Of the 50 cases who were aware of their HIV status, 46 (92.0%) were currently on ART.

### Substance use

Table 2 shows that the median time since the participants started using drugs was 12 months (IQR = 6–36). The majority (93.1%) reported “smoking” as the method of their first drug use and that they were firstly introduced to drugs by their friends or co-workers (79.7%). Methamphetamine (Yama or ice) was the most commonly used drugs in the past 3 months (95.1%), and only 1.8% reported having used injecting drugs in their lifetime. The use of other substances was also common, with 31.5% reported alcohol drinking at least four times per week, and 33.0% reported binge drinking (drinking at least 5 units of alcohol in a single session) at least on 4 days per week in the past 3 months. Almost half (44.8%) reported smoking at least 100 cigarettes in their lifetime. The proportion of those who reported having used injecting drugs in their lifetime was significantly higher (10.6% vs. 1.2%) in HIV-positive group compared to that in HIV-negative group.

**Table 2:** Characteristics of substance use among HIV-positive and HIV-negative PWUD

Drug use characteristics	Total (*n* = 1367)	HIV test result		
		Positive (*n* = 78)	Negative (*n* = 1289)	
	*n* (%)	*n* (%)	*n* (%)	*P*-value^*^
Method of first illicit drug use				0.81
Injecting	3 (0.2)	0 (0.0)	3 (0.2)	
Smoking	1273 (93.1)	75 (96.2)	1198 (92.9)	
Sniffing or snorting	76 (5.6)	73 (5.7)	3 (3.8)	
Swallowing/drinking	15 (1.1)	15 (1.1)	0 (0.0)	
First-time drug use was introduced to drugs by:				0.60
Myself	206 (15.1)	10 (12.8)	196 (15.2)	
Friends/co-workers	1089 (79.7)	66 (84.6)	1023 (79.4)	
Sexual partners	58 (4.2)	2 (2.6)	56 (4.3)	
Stranger (forced by)	13 (1.0)	0 (0.0)	13 (1.0)	
Used illicit drugs in the past 3 months	1053 (77.8)	66 (84.6)	987 (77.4)	0.13
Type of illicit drugs commonly used in the past 3 months				
Yama/ice (methamphetamine)	1014 (95.1)	63 (62.8)	951 (95.1)	0.90
Ecstasy	59 (5.5)	5 (7.6)	54 (5.4)	0.46
Inhalants	48 (4.5)	7 (10.6)	41 (4.1)	0.06
Cannabis	35 (3.3)	5 (7.6)	30 (3.0)	0.08
Having injected illicit drugs in lifetime	19 (1.8)	7 (10.6)	12 (1.2)	< 0.001
Alcohol drinking ≥4 times per week	429 (31.5)	23 (29.5)	406 (31.6)	0.70
Binge drinking ≥4times per week	374 (33.0)	354 (32.9)	20 (34.5)	0.80
Smoked at least 100 cigarettes in lifetime	611 (44.8)	32 (41.0)	579 (45.0)	45.0

HIV, human immunodeficiency virus; PWUD, people who use drugs

^*^Chi-square test (or Fisher’s exact test when a cell count was smaller than 5) was used

### Sexual behaviors

The majority of the participants (92.4%) reported being sexually active in the past 3 months, with a median number of sex partners of 1.0 (IQR = 1.0–3.0). As presented in Table 3, only 26.3% reported always using condoms with any types of partners, and 39.1% reported having sexual intercourse when a partner was intoxicated in the past 3 months. Of those who reported having sex with partners not in exchange for money or goods (*n* = 507), 23.2% reported always using condoms with the non-commercial partners in the past 3 months. Of the total participants, 38.5% reported having sex in exchange for money or goods in the past 3 months; of whom, 40.5% reported always using condoms with the commercial partners over the same period. Despite their overall high HIV risk, only 22.1% perceived that their HIV risk was higher compared to that in the general population. Compared to the HIV-negative group, a significantly higher proportion of HIV-positive participants reported always using condoms with any types of partners (39.6% vs. 25.6%) and having sex in exchange for money or gifts (58.5% vs. 37.3%) in the past 3 months. A significantly higher proportion of HIV-positive participants perceived that their HIV risk was higher compared to that in the general population (46.2% vs. 20.6%).

**Table 3:** Sexual behaviors and perceived HIV risk among HIV-positive and HIV-negative PWUD

Sexual behaviors in the past 3 months	Total (*n* = 1367)	HIV test result		
		Positive (*n* = 78)	Negative (*n* = 1289)	
	*n* (%)	*n* (%)	*n* (%)	*P*-value^*^
Had sexual intercourse	1263 (92.4)	75 (96.2)	1188 (92.2)	0.20
Median number of sex partners (IQR)	1.0 (1.0–3.0)	1.0 (0.0–0.4)	1.0 (1.0–3.0)	0.60
Always used condoms with any type of partners	254 (26.3)	21 (39.6)	233 (25.6)	0.02
Had sex when partner was intoxicated	375 (39.1)	22 (43.1)	353 (38.8)	0.54
Had sex with non-commercial partners (*n* = 966)	507 (52.5)	30 (56.6)	477 (52.3)	0.54
Always used condoms with non-commercial partners (*n* = 507)	119 (23.2)	10 (32.3)	109 (22.7)	0.22
Had sex in exchange for money or gifts (*n* = 966)	372 (38.5)	31 (58.5)	341 (37.3)	0.002
Always used condoms with commercial partners (*n* = 372)	150 (40.5)	15 (48.4)	135 (39.8)	0.35
Perceived HIV risk compared to the general population				< 0.001
Higher	301 (22.1)	36 (46.2)	265 (20.6)	
About the same	438 (32.2)	24 (30.8)	414 (32.2)	
Lower	219 (16.1)	7 (9.0)	212 (16.5)	
Don’t know	404 (29.7)	11 (14.1)	393 (30.6)	

HIV, human immunodeficiency virus; IQR, interquartile range; PWUD, people who use drugs

^*^Chi-square test was used for categorical variables and Mann-Whitney U test for continuous variables

### Sexually transmitted infections

As shown in Tables 4, 3.4% of participants were tested positive for syphilis, and 33.6% reported having had at least one STI symptom in the past 12 months. The most commonly reported symptoms included abnormal urethral discharge (79.5%), followed by having cuts or sores (15.6%) and swelling (15.2%) around the genital area. Compared to HIV-negative group, a significantly higher proportion of HIV-positive participants reported having an STI symptom in the past 12 months (48.7% vs. 33.5%) and having cuts or sores around the genital area (26.3% vs. 14.7%). However, a significantly lower proportion of HIV-positive participants reported having abnormal urethral discharge compared to that of HIV-negative group (65.8% vs. 80.7%).

**Table 4:** Sexually transmitted infections among HIV-positive and HIV-negative PWUD

STI symptoms in the past 12 months	Total (*n* = 1367)	HIV test result		
		Positive (*n* = 78)	Negative (*n* = 1289)	
	*n* (%)	*n* (%)	*n* (%)	*P*-value^*^
Tested positive for syphilis	47 (3.4)	5 (6.4)	42 (3.3)	0.14
Had an STI symptom	470 (34.4)	38 (48.7)	432 (33.5)	0.006
Cuts or sores around genital area	73 (15.6)	10 (26.3)	63 (14.7)	0.04
Swelling around genital area	71 (15.2)	5 (13.2)	66 (15.3)	0.72
Abnormal urethral discharge	372 (79.5)	25 (65.8)	347 (80.7)	0.02
Symptoms around the anus	23 (4.9)	4 (10.5)	19 (4.4)	0.10
Symptoms in the mouth or throat	40 (10.0)	5 (13.2)	42 (9.8)	0.51
Received treatment for most recent STI	373 (80.2)	32 (84.2)	341 (79.9)	0.52

HIV, human immunodeficiency virus; STI, PWUD, people who use drugs; sexually transmitted infections

^*^Chi-square or Fisher’s exact test was used as appropriate

### Access to community-based HIV services

Table 5 shows that 54.5% of the study participants reported having received some form of community-based HIV services in the past 6 months. The services included condom and lubricant distribution (81.7%), HIV/syphilis testing (60.0%), HIV education (53.3%), harm reduction (14.6%), drop-in services (13.6%), legal support (4.5%), and drug rehabilitation (1.3%). Compared to HIV-negative group, a significantly higher proportion of HIV-positive participants reported having received community-based HIV services in the past 6 months (84.6% vs. 52.7%).

**Table 5:** Access to community-based HIV services among HIV-positive and HIV-negative PWUD

Access to community-based HIV services in the past 6 months	Total (*n* = 1367)	HIV test result		
		Positive (*n* = 78)	Negative (*n* = 1289)	
	*n* (%)	*n* (%)	*n* (%)	*P*-value^*^
Received community-based HIV services	745 (54.5)	66 (84.6)	679 (52.7)	< 0.001
HIV education material distribution	251 (53.3)	28 (58.8)	223 (53.2)	0.92
Condom and lubricant distribution	385 (81.7)	39 (75.0)	346 (82.6)	0.18
Needle and syringe distribution	69 (14.6)	4 (7.7)	65 (15.5)	0.13
HIV/syphilis testing services	283 (60.0)	27 (51.9)	256 (61.1)	0.20
Legal support services	21 (4.5)	1 (1.9)	20 (4.8)	0.35
Drop-in services	64 (13.6)	7 (13.5)	57 (13.6)	0.98
Rehabilitation services	6 (1.3)	0 (0.0)	6 (1.4)	0.39

HIV, human immunodeficiency virus; PWUD, people who use drugs

^*^Chi-square or Fisher’s exact test was used as appropriate

### Factors associated with HIV infection

Independent factors associated with HIV infection are shown in Table 6. After adjustment for other covariates in the multivariable logistic regression model, HIV infection remained significantly associated with being in the age group of ≥35 (AOR = 2.34, 95% CI = 1.04–6.11), having lower level of formal education of ≤ 6 years (AOR = 2.26, 95% CI = 1.04–5.15), living on the streets (AOR = 2.82, 95% CI = 1.10–7.23), perception that their HIV risk was higher as compared to that of the general population (AOR = 3.18, 95% CI = 1.27–8.62), having used injecting drugs in lifetime (AOR = 3.8, 95% CI = 1.36–4.56), and having cuts or sores around the genital area in the past 12 months (AOR = 3.42, 95% CI = 1.09–6.33).

**Table 6:** Factors associated with HIV infection among PWUD in a multivariate logistic regression model

Variables in the final model	AOR (95% CI)	*P*-value
Age group		
< 25	Reference	
25–34	0.91 (0.37–2.26)	0.84
≥ 35	2.34 (1.04–6.11)	0.04
Level of formal education attained		
High school or higher (≥10 years)	Reference	
Secondary school (7–9 years)	1.07 (0.41–2.77)	0.89
Primary (0–6 years)	2.26 (1.04–5.15)	0.04
Living situation		
With family	Reference	
On the streets (homeless)	2.82 (1.10–7.23)	0.03
In own dwelling	1.73 (0.67–4.44)	0.26
With friends	0.94 (0.27–3.29)	0.92
Perceived HIV risk compared to the general population		
Lower	Reference	
About the same	1.56 (0.52–4.70)	0.43
Higher	3.18 (1.27–8.62)	0.004
Having used injecting drugs in lifetime		
No	Reference	
Yes	3.81 (1.36–4.56)	0.001
Having cuts or sores around the genital area in the past 12 months		
No	Reference	
Yes	3.48 (1.09–6.33)	0.008

Abbreviations: AOR, adjusted odds ratio; CI, confidence interval; HIV, human immunodeficiency virus; PWUD, people who use drugs

^*^Age, gender, marital status, education level, and variables associated with HIV infection in the bivariate analyses at a level of p < 0.05 were simultaneously included in the model

## Discussion

This paper presents crucial data regarding the prevalence of HIV and its risk factors among Cambodian PWUD sampled in the most recent national IBBS conducted in 2017. The findings present useful information related to the profile of substances used by the participants. Methamphetamines were particularly prominent in the profile of substances recently consumed (95.1%), while ecstasy, cannabis, and other inhalants feature less, having been used by 3.0 to 5.4% of the participants. These findings concur with previous studies that have reported the ubiquity of methamphetamines in Cambodia [19–22]. As expected, most PWUD used drugs by way of smoking, while fewer others snorted or swallowed them. A small proportion (0.2%) had a history of drug injection in their lifetime, even though this was more than 12 months prior to the survey.

Overall, we found that the prevalence of HIV was 5.7%, about 10 times higher than the prevalence in the general adult population [6]. This finding confirms that non-injecting drug users are highly vulnerable to HIV infection. Independent risk factors of HIV infection identified in this study included older age, lower level of education, homelessness, perception of high level of HIV risk, previous drug injection, and STI symptoms. These findings warrant further discussion.

To start with, these findings suggest that older age may be related to the risk of HIV infection. This is in contrast to other studies in China and South Africa, which did not find a relationship between HIV infection and age among methamphetamine users [23, 24]. However, a previous study among transgender in Cambodia reported that older age increased the risk of HIV infection [25]. Bivariate findings in our study suggested that duration of use is predictive of HIV infection, and the duration was also identified as predictive of acquisition of HIV and hepatitis C virus infections in China [23]. As PWUD continue to consume drugs, the cumulative chances of HIV acquisition would be expected to increase over time, especially if these drugs are consumed in environments that promote risky sexual practices. Our study suggests that this could be the case. For instance, 31.5% of the participants reported alcohol drinking at least four times per week, and 33.0% reported binge drinking at least on 4 days per week in the past 3 months. Furthermore, the prevalence of transactional sex and STIs in our sample was considerably high, and genital ulcers were independently predictive of HIV infection. Stimulants such as methamphetamine and ecstasy are particularly associated with risky sexual practices such as unprotected or transactional sex with multiple partners [7, 26, 27] through enhanced sexual desire [28].

Not surprisingly, history of having used injecting drugs was associated with higher likelihood of being diagnosed with HIV. This is consistent with assertions that injecting drug use is a potential risk factor for HIV due to the risk of sharing contaminated needles [7]. Together with the above assertions, this finding suggests that users of stimulants predominantly expose themselves to HIV sexually, while drug injection can also determine HIV acquisition among those who transition to injecting, even temporarily. The finding that a history of drug injection tripled the odds of HIV infection provides a compelling reason to reach out to people who are currently using drugs by routes other than injection, as some of them may have injected in the past, often over a year ago as was the case in our study. Taking the history of lifetime drug injection provides a programmatically useful way of stratifying risk among current PWUD. Stratifying profiles of HIV risk could be an important and cost-effective strategy for ensuring that every HIV infection is identified within the current 90–90-90 global goals and the Cambodia’s 3.0 strategy.

A quarter of the respondents in this study perceived that they were at higher risk of HIV infection compared to the general population. HIV was more likely to occur among these PWUD who perceived that they were at high HIV risk, perhaps based on their sexual behaviors, which is not necessarily surprising. Perception of risk has been shown to influence the uptake of HIV prevention interventions [29], and to act in association with contextual factors to influence the risk of HIV infection [30]. As noted in Table 5, the majority of the respondents in this survey had been reached with a range of community-based HIV programs, including risk reduction education interventions and education related to their risks. It is therefore reasonable to expect that these populations would be aware of risks of HIV infection. The importance of our findings, however, is that participants who clearly thought they were at high risk might have not been taken measures to reduce those risks, leading to their acquisition of HIV infection. This discordancy might be explained by a lack of self-efficacy to reduce their exposure to HIV, or a range of external determinants that could reduce their ability to manage their exposure to HIV despite being aware of risks.

Determinants of health behaviors such as low self-efficacy, low motivation, and external barriers have been shown to prevent PWID in India from accessing health services [31]. Data showing the complex interplay between recognition of vulnerability, rationalization of risk, and inaction to protect oneself from HIV despite high-risk perception has been reported among heterosexual women in Kenya and South Africa [30]. Therefore, it is not entirely implausible that a range of barriers prevented PWUD who thought that they were at high risk from adopting safer behaviors. However, the validity of this speculation will need to be explored specifically among this sub-population who perceive higher risk of HIV compared to the general population. Previous studies conducted in Cambodia have highlighted that the lack of an enabling environment perpetuated by legal and social factors impedes PWUD’s access to health service including HIV prevention and care, and thus threat efforts to control HIV among PWUD [14, 32]. Future research should seek to understand how perceived high HIV risk is actioned or otherwise rationalized, leading to inaction, among PWUD in our study setting. This is particularly important given that more than one-third (35.2%) of the HIV-positive cases were not aware of their status prior to the survey.

An important finding from this study is that PWUD who lived on the streets were more likely to be infected with HIV. Previous studies have shown the impact of lack of housing on risky substance use and sexual behaviors among PWUD, including those who used amphetamine and ecstasy [33–35]. Structural disadvantages, such as homelessness, are known to encourage high-risk behaviors by providing environments for experimentation among street-dwellers [36]. This finding is important as it provides an additional and practically useful way in which to stratify profiles of risk among PWUD, while suggesting that structural shelter accommodation could be useful. At the same time, programs should place a higher emphasis on homeless PWUD, ensuring that outreach workers provide intensive education and support to this population.

Finally, an expected finding from this study was the observation that lower education predicted higher risk of HIV infection. Previous studies in Cambodia have shown that both substance use and HIV infection are associated with lower levels of education [25, 37]. This finding adds additional impetus for reaching PWUD with information and education regarding harms of drug use, with relevant and easy-to-access materials, or through peer-led approaches, prioritizing those that have low literacy and education levels.

### Limitations of the study

In interpreting the findings from this survey, several limitations should be borne in mind. First, this was a cross-sectional study which provides a snapshot of the prevalence of HIV and related risk behaviors, and does not capture the dynamic way in which profiles and modes of drug use change in the life course [38]. Second, this survey was conducted in 12 provinces with a high burden of HIV and drug use, leaving out some 13 other provinces with lower prevalence of both HIV and drug use. This may affect the generalizability of the study findings at the national level. Third, self-reporting measures were used to collect a number of correlates, which are likely to be affected by social desirability bias known to affect reporting of sensitive drug use and sexual behaviors [39]. Finally, this survey used incentives in the RDS sampling to recruit the participants, which may have affected PWUD’s genuine motivation to participate. However, we anticipate that the resultant motivational bias had minimal impact on our overall findings.

## Conclusions

Strengthening the strategic information related to illicit drug use is one of the five objectives of the Cambodian National Strategic Plan on Harm Reduction [5]. This study reports an HIV prevalence of 5.7% among 1367 PWUD recruited from the capital city and 11other major provinces out of the 25 provinces in the countries. A range of factors independently associated with HIV infection are reported; these included older age, lower education, homelessness, high-risk perception, history of drug injection, and a recent history of genital ulcers or sores. In addition, the study documents the predominance of methamphetamines in the profile of non-injecting drugs in the study context. This is an important addition to the national strategic information related to illicit drug use, as global studies related to amphetamine-type stimulants (ATS) use tend to focus on men who have sex with men and in high-income countries, with relatively limited exploration of the use in low- and middle-income countries [40]. Given the findings of this study and to achieve the national strategic goals of halting the HIV epidemic among PWUD, our findings suggest that stratification of risk based on socio-economic profiles and drug injection history can enhance programs to identify unidentified people living with HIV.

## Data Availability

Data used for this study can be accessed upon request from the Principal Investigator (Dr. Siyan Yi) at siyan@doctor.com.

## References

[1] The United Nations Office on Drugs and Crime (2016). World drug report.

[2] Degenhardt L, Whiteford HA, Ferrari AJ, Baxter AJ, Charlson FJ, Hall WD (2013). Global burden of disease attributable to illicit drug use and dependence: findings from the global burden of disease study 2010. Lancet..

[3] Whiteford HA, Degenhardt L, Rehm J, Baxter AJ, Ferrari AJ, Erskine HE (2013). Global burden of disease attributable to mental and substance use disorders: findings from the global burden of disease study 2010. Lancet..

[4] Chhea C, Heng S, Tuot S. National Popualtion size estimtion, HIV relted risk behaviors, HIV prevalence among people who use drugs in Cambodia in 2012. Phnom Penh: National Center for HIV/AIDS, dermatology and STD; 2014.

[5] National AIDS Authority. Cambodia Country Progress Report. In: Monitoring Progress Towards the 2011 UN Political Declaration on HIV and AIDS. Phnom Penh: National AIDS Authority; 2015.

[6] Vun MC, Fujita M, Rathavy T, Eang MT, Sopheap S, Sovannarith S (2014). Achieving universal access and moving towards elimination of new HIV infections in Cambodia. J Int AIDS Soc.

[7] Tavitian-Exley I, Vickerman P, Bastos FI, Boily MC (2015). Influence of different drugs on HIV risk in people who inject: systematic review and meta-analysis. Addiction..

[8] Degenhardt L, Peacock A, Colledge S, Leung J, Grebely J, Vickerman P (2017). Global prevalence of injecting drug use and sociodemographic characteristics and prevalence of HIV, HBV, and HCV in people who inject drugs: a multistage systematic review. Lancet Glob Health.

[9] Jones JD, Mogali S, Comer SD (2012). Polydrug abuse: a review of opioid and benzodiazepine combination use. Drug Alcohol Depend.

[10] Leri F, Bruneau J, Stewart J (2003). Understanding polydrug use: review of heroin and cocaine co-use. Addiction..

[11] Kermode M, Songput CH, Sono CZ, Jamir TN, Devine A (2012). Meeting the needs of women who use drugs and alcohol in north-East India - a challenge for HIV prevention services. BMC Public Health.

[12] United Nations High Commissioner for Human Rights. Law on Control of Drugs. Phnom Penh: United Nations High Commissioner for Human Rights;1996.

[13] Mun P, Yi S, Dousset J, Chann N, Tuot S, Chhim S, Chhoun P (2017). National integrated biological and behavioral survey and population size estimation among people who use and inject drugs in Cambodia. Phomn Penh: National Center for HIV/AIDS, dermatology and STD.

[14] Yi S, Tuot S, Chhoun P, Pal K, Choub SC, Mburu G (2016). Prevalence and correlates of psychological distress among drug users in Phnom Penh, Cambodia. Int J Drug Policy..

[15] Mun P, Tuot S, Chhim S, Chhoun P, Ly C, Paldyla K, et al. Integrated Biological and Behavioral Survey among Transgender Women in Cambodia. Phnom Penh: National Center for HIV/AIDS, Dermatology and STD;2016.

[16] Mun P, Chhim S, Chhoun P, Tuot S, Ly C, Dionisio J, et al. National population size estimation, HIV related risk behaviors, and HIV Prevalence among men who have sex with men in Cambodia in 2014. Phnom Penh: National Center for HIV/AIDS, Dermatology and STD; 2016.

[17] Yi S, Chhoun P, Brant S, Kita K, Tuot S. The Sustainable Action against HIV and AIDS in Communities (SAHACOM): End-of-Project Evaluation. Phnom Penh: KHANA;2014.

[18] National Institute of Statistics, Directorate general for health, ICF international. Cambodia demographic and health survey 2014. Phnom Penh: National Institute of Statistics; 2015.

[19] Thomson N, Thomson N (2010). Detention as treatment: detention of methamphetamine users in Cambodia.

[20] McKetin R, Kozel N, Douglas J, Ali R, Vicknasingam B, Lund J (2008). The rise of methamphetamine in southeast and East Asia. Drug Alcohol Rev..

[21] Kulsudjarit K (2004). Drug problem in southeast and Southwest Asia. Ann N Y Acad Sci.

[22] Mburu G, Ngin C, Tuot S, Chhoun P, Pal K, Yi S (2017). Patterns of HIV testing, drug use, and sexual behaviors in people who use drugs: findings from a community-based outreach program in Phnom Penh, Cambodia. Addict Sci Clin Pract.

[23] Bao YP, Liu ZM, Lian Z, Li JH, Zhang RM, Zhang CB, Hao W, Wang XY, Zhao M, Jiang HF (2012). Prevalence and correlates of HIV and HCV infection among amphetamine-type stimulant users in 6 provinces in China. J Acquir Immune Defic Syndr.

[24] Gouse H, Joska JA, Lion RR, Watt MH, Burnhams W, Carrico AW, Meade CS (2016). HIV testing and sero-prevalence among methamphetamine users seeking substance abuse treatment in Cape Town. Drug Alcohol Rev.

[25] Chhim S, Ngin C, Chhoun P, Tuot S, Ly C, Mun P (2017). HIV prevalence and factors associated with HIV infection among transgender women in Cambodia: results from a national integrated biological and behavioral survey. BMJ Open.

[26] Plankey MW, Ostrow DG, Stall R, Cox C, Li X, Peck JA, Jacobson LP (2007). The relationship between methamphetamine and popper use and risk of HIV seroconversion in the multicenter AIDS cohort study. J Acquir Immune Defic Syndr.

[27] Degenhardt L, Mathers B, Guarinieri M, Panda S, Phillips B, Strathdee SA (2010). Meth/amphetamine use and associated HIV: implications for global policy and public health. Int J Drug Policy..

[28] Volkow ND, Wang GJ, Fowler JS, Telang F, Jayne M, Wong C (2007). Stimulant-induced enhanced sexual desire as a potential contributing factor in HIV transmission. Am J Psychiatry.

[29] Warren EA, Paterson P, Schulz WS, Lees S, Eakle R, Stadler J (2018). Risk perception and the influence on uptake and use of biomedical prevention interventions for HIV in sub-Saharan Africa: a systematic literature review. PLoS One.

[30] Corneli AL, McKenna K, Headley J, Ahmed K, Odhiambo J, Skhosana J (2014). A descriptive analysis of perceptions of HIV risk and worry about acquiring HIV among FEM-PrEP participants who seroconverted in Bondo, Kenya, and Pretoria, South Africa. J Int AIDS Soc.

[31] Chakrapani V, Velayudham J, Shunmugam M, Newman PA, Dubrow R (2014). Barriers to antiretroviral treatment access for injecting drug users living with HIV in Chennai, South India. AIDS Care.

[32] Tuot S, Ngin C, Pal K, Sou S, Sawez G, Morgan P, et al. How understanding and application of drug-related legal instruments affects harm reduction interventions in Cambodia: a qualitative study. Harm Reduct J. 2017;14(1):39. 10.1186/s12954-017-0167-9. 10.1186/s12954-017-0167-9 PMC5477156 28629463

[33] Cheng T, Johnston C, Kerr T, Nguyen P, Wood E, DeBeck K (2016). Substance use patterns and unprotected sex among street-involved youth in a Canadian setting: a prospective cohort study. BMC Public Health.

[34] Gleghorn AA, Marx R, Vittinghoff E, Katz MH (1998). Association between drug use patterns and HIV risks among homeless, runaway, and street youth in northern California. Drug Alcohol Depend.

[35] Damon W, McNeil R, Milloy MJ, Nosova E, Kerr T, Hayashi K (2019). Residential eviction predicts initiation of or relapse into crystal methamphetamine use among people who inject drugs: a prospective cohort study. J Public Health.

[36] Feng C, DeBeck K, Kerr T, Mathias S, Montaner J, Wood E (2013). Homelessness independently predicts injection drug use initiation among street-involved youth in a Canadian setting. J Adolesc Health.

[37] Weissman A, Ngak S, Srean C, Sansothy N, Mills S, Ferradini L (2016). HIV prevalence and risks associated with HIV infection among transgender individuals in Cambodia. PLoS One.

[38] Hser YI, Longshore D, Anglin MD (2007). The life course perspective on drug use: a conceptual framework for understanding drug use trajectories. Eval Rev.

[39] Latkin CA, Vlahov D, Anthony JC (1993). Socially desirable responding and self-reported HIV infection risk behaviors among intravenous drug users. Addiction..

[40] Colfax G, Santos GM, Chu P, Vittinghoff E, Pluddemann A, Kumar S (2010). Amphetamine-group substances and HIV. Lancet..

